# Catarrhal Fever

**Published:** 1891-11

**Authors:** V. D. Lockhart

**Affiliations:** Homer, Ga.


VOL. VIII. NOVEMBER, 1891. NO. 9.

TO CONTRIBUTORS.

Articles for publication must reach this office not later than 15th inst.; and are expected to be published exclusively in this JOURNAL. Extra copies of JOURNAL furnished contributors when requested. Reprints by special arrangement. Publication of an article does not necessarily imply endorsement of the views therein expressed.

ATLANTA MEDICAL AND SURGICAL JOURNAL, Post-office box 431.

### Original Communications.

### CATARRHAL FEVER.

BY V. D. LOCKHART, M. D., HOMER, GA.

This disease embraces two stages, the dry and the moist, and the indications for treatment are somewhat different in each.

To relieve the fever and coryza of the first stage antifebrin is a good remedy. It may be combined with quinine.

℞. Antifebrin, gr. viij—x.
Quinine, gr. v.

M. This will generally insure rest and a moist skin.

A purgative,

℞. Hydrarg. chlor. mitis., gr. v.
Ipecac, gr. j.
Rhei pulv., gr. viij.

M. One dose, to be followed by a dose of Epsom salts or castor oil if needed.

Order a bowl of hot water, add a few drops oil turpentine; let the patient inhale the vapor, a shawl or blanket thrown over the head to confine the steam. This often affords much relief. If the throat be sore apply turpentine to the fauces with a swab.

After the purgative has acted give,

℞. Tinct. aconiti rad., ʒj.

Vini antimonii, ʒijss.

Spt. ether. nitros., ʒvj.

Liquor ammon. acetatis, q. s. ad. ℥jv.

One-half teaspoon- to teaspoonful every two to four hours as indicated. Children four or five years of age twenty drops. Opiates are not good for this stage, but if there is much restlessness a dose of Dover's powder may be given. Antikamnia is better. Mustard should be used as a counter-irritant. One part of ground mustard to two of flour. It should be applied frequently during the disease, and when the mustard is not on, a poultice of wheat bran or cloths wrung out of warm water, and over it a layer of oiled silk. Quinine in moderate doses three times a day. The temperature of the room should be kept pleasant. Under this treatment the cough will become loose, fever will subside and dyspnœa and soreness of the chest cease in a day or two. Then give,

℞. Syrup scillæ,

Syrup senagæ,

Syrup tolut.,

Tinct. opii. camphorat., aa ℥j.

Ammonii chlorid., ʒij.

M. Sig.—Teaspoonful every three or four hours. Digitalis comes in well and it may be added to the syrup as indicated.

In small children prompt and efficient measures are often needed to relieve the dyspnœa and other threatening symptoms. Give fluid extract ipecac in doses sufficient to insure free emesis. This may be necessary at intervals for several days, but it must not be given so as to keep the child nauseated, as this interferes with the measures of support, which are important, especially if the child have pertussis. In this disease we must support the strength by using stimulants and rich, nourishing food, and they are indicated early. Besides the usual treatment indicated above the following is a good prescription for *whooping-cough :*

℞. Ext. cannabis indicæ, gr. xv.

Ext. belladonnæ, gr. viij.

Alcohol,

Glycerin, ad. ℥jss.

M. Sig.—Four or five drops to a child one year old; one of two years old, five to a eight drops three or four times a day.

